# *Abiotrophia defectiva* Endocarditis: An Easy Miss

**DOI:** 10.5811/cpcem.2017.3.33126

**Published:** 2017-07-14

**Authors:** Erica Escarcega, Caitlin Trovato, Osamudiamen Idahosa, John Gillard, Holly Stankewicz

**Affiliations:** * St. Luke’s University Hospital, Department of Emergency Medicine, Bethlehem, Pennsylvania; † St. Luke’s University Hospital, Department of Critical Care Medicine, Bethlehem, Pennsylvania

## Abstract

Subacute endocarditis often presents with an indolent course. A potentially lethal form generated by infection with *Abiotrophia defectiva* may be easily overlooked early in its presentation. This report discusses the case of an 18-year-old male discovered to have severe endocarditis after presenting to the emergency department with the chief complaint of foot pain.

## INTRODUCTION

*Abiotrophia defectiva*, once classified as a nutritionally variant strain of streptococci, is a rare but important cause of infective endocarditis with potentially devastating consequences due to its high rates of embolization, bacteriological failure and mortality.^1^,^2^ This little-known bacteria is part of the normal oral flora making dental procedures a commonly implicated source of infection.^2^,^3^,^4^ Unfortunately, this bacteria is often pleomorphic on Gram stain and is difficult to isolate, requiring specialized media and, therefore, may be the cause of some cases of culture-negative endocarditis.^2^,^3^ Here we describe a case of subacute bacterial endocarditis in a previously healthy young male with history of bicuspid aortic valve.

## CASE REPORT

An 18-year-old male with history of bicuspid aortic valve presented to the emergency department (ED) the day after his senior prom with the chief complaint of right foot pain that had been gradually worsening over the prior three days. He had awoken at 4:30 am that morning and found that he could no longer bear weight on the right foot due to the intensity of the pain and had been using an old set of crutches to ambulate around his home. The patient indicated that the pain was primarily over the dorsal aspect of the foot, radiated up the back of the calf and was worse with bearing weight and movement. He had taken ibuprofen at home with no improvement in his symptoms. He denied any recent strenuous activity or injury to the foot, but had been dancing at his prom the previous night. He had been treated for plantar fasciitis in both feet by his podiatrist and had received a cortisone shot in the left foot three weeks earlier. Patient had also been having symptoms of fatigue, night sweats and fever for which his primary care physician had prescribed levofloxacin. He had completed one 10-day course 2–3 weeks prior to his presentation with only minimal improvement in his symptoms and had been started on a second 10-day course. The patient had been afebrile for the prior week, but he had continued to have problems with fatigue and night sweats and had recently developed exertional dyspnea. He denied intravenous drug use or recent dental procedures.

On initial examination, the patient was well appearing, but tachycardic at 118 beats per minute (bpm) with a grade 2/6 systolic murmur. He had normal breath sounds, was not tachypneic, and had a normal oxygen saturation. On examination of the right foot and ankle, he had tenderness over the dorsum of the foot and pain with range of motion of the ankle. Distal perfusion and sensation of the foot were intact. There were no overlying skin changes. The classic skin findings of endocarditis, including Janeway lesions, Osler nodes and splinter hemorrhages, were not present. A radiograph of the right foot was negative for fractures or dislocations. Basic labs including a blood count and basic metabolic panel were drawn that were significant only for an unexplained anemia with a hemoglobin of 8.9 g/dL. The patient was given a 1 L bolus of normal saline, but remained tachycardic on re-examination with a heart rate as high as 134 bpm. His electrocardiogram was otherwise normal. The decision was made to draw a D-dimer, which came back elevated at 721 μg/L. After a discussion with the patient and his mother, a computed tomography (CT) with contrast to rule out pulmonary embolism (PE) was ordered. Although negative for PE, his CT had concerning findings including an ascending thoracic aortic aneurysm measuring 4.1 cm, as well as ground-glass densities of the lungs. The patient was admitted to the hospital for an urgent echocardiogram, which revealed severe endocarditis affecting both the mitral and aortic valves (Image). Blood cultures were drawn and he was started on vancomycin and ceftriaxone.

The patient remained hemodynamically stable; however, three days after his admission, he developed multiple embolic phenomena manifesting as a left frontal infarct as well as an acute thrombus to the right internal jugular. After a negative magnetic resonance image of the ankle, a duplex ultrasound revealed the presence of an acute deep vein thrombosis of the right posterior tibial vein. Blood cultures were positive for *Abiotrophia defectiva*, but due to the difficulty in growing the organism the sample was sent to an outside facility and sensitivities were delayed. When sensitivities resulted, the organism was found to be sensitive to the empiric regimen. Notably, the organism was also sensitive to levofloxacin. The use of this antibiotic prior to his presentation may have contributed to his relatively benign initial presentation. The patient underwent bovine aortic and mitral valve replacements after which he was extubated and weaned off vasopressor and inotropic support. On post operative day 1, he developed a severe systemic inflammatory state with multi-organ system failure requiring re-intubation, escalation of vasopressors and inotropes, continuous renal replacement therapy and urgent venoarterial extracorporeal membrane oxygenation support due to refractory shock. A bedside echocardiogram revealed biventricular failure with ejection fraction of 5%. A biventricular assist device was later inserted, and approximately two months later the patient underwent successful heart transplantation.

## DISCUSSION

This case highlights the need for increased awareness of the potentially indolent presentation as well as the severity, morbidity and mortality related to *Abiotrophia defectiva* endocarditis. There are case reports of *Abiotrophia defectiva* endocarditis affecting both the young and the old, and patients with underlying heart disease and even those without.^2^,^4^,^5^ This diagnosis is difficult as patients often present with signs more typical of subacute endocarditis that are often nonspecific, including low-grade fever, weakness, fatigue and myalgias.^6^ Classically taught manifestations of endocarditis such as Roth’s spots, Janeway lesions, Osler nodes and splenomegaly are less common in children regardless of the bacterial etiology.^6^ Penicillins in combination with gentamicin are recommended as first-line therapy for*Abiotrophia* endocarditis by American Heart Association guidelines.^3^ However, although there is little resistance reported to gentamicin or vancomycin, this potentially deadly bacteria has shown resistance to penicillins.^2^,^7^ Making treatment even more difficult, *Abiotrophia defectiva* has been found to have a slow metabolic rate, which is believed to contribute to the higher rate of treatment failures.^4^

Population Health Research Capsule

What do we already know about this clinical entity?

Endocarditis is a difficult diagnosis to make in the emergency department. Patients may present with classic signs and symptoms or may have a more indolent course with nonspecific symptoms.

What makes this presentation of disease reportable?

This is a case of a young male that presented with a very benign complaint despite having severe disease. Investigation of unexplained tachycardia led to his eventual diagnosis.

What is the major learning point?

Subacute endocarditis often presents with vague symptomatology including musculoskeletal complaints.

How might this improve emergency medicine practice?

Considering subacute endocarditis in patients with vague presentations and unexplained tachycardia may uncover cases that may be otherwise missed.

## CONCLUSION

Devastating complications, such as subarachnoid hemorrhage from mycotic cerebral aneurysms and heart failure, have been reported in association with this form of endocarditis, highlighting the need for keeping a high degree of suspicion when treating these patients who, early on, may have very mild presentations.^2^,^8^

## Figures and Tables

**Image:**
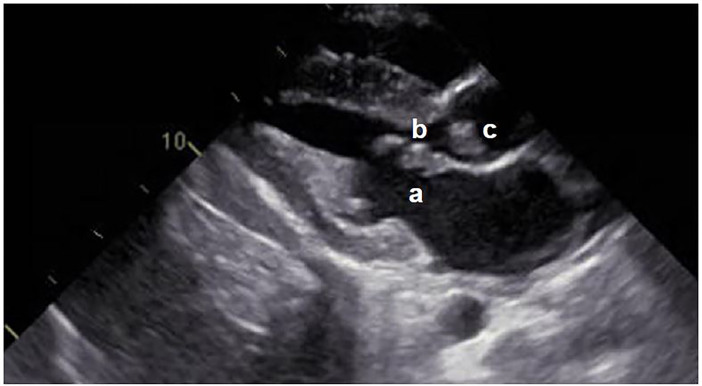
Transthoracic echocardiogram showing focal thickening of the anterior leaflet of the mitral valve (a) with a large perforation (b), as well as increased thickness of the aortic valve with vegetations (c).
